# Supplementary material for: Mutations in PCYT2 disrupt etherlipid biosynthesis and cause a complex hereditary spastic paraplegia
Source: Brain. 2019 Oct 22;142(11):3382–97. doi: 10.1093/brain/awz291 (PMC6821184; doi:10.1093/brain/awz291)
Supplement: awz291_Supplementary_Data [file awz291_supplementary_data.zip › awz291-Suppl_data/Supplementary_Data4.zip!/Supplementary information 3 - fibroblast lipidomics.zip!/Fibroblast lipidomics/Controle vs Patient1/Statistical plots and data/PE[O] species/Boxplots all.pdf]

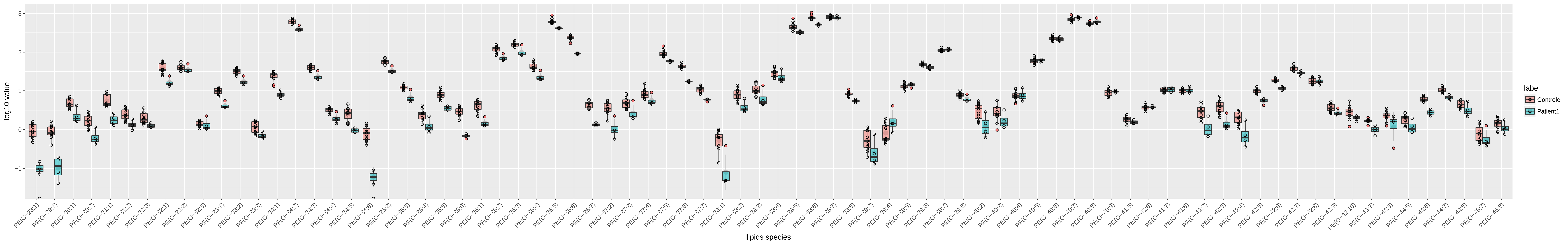

log10 value
3
2
1
0
-1
lipids species
PE(O−28:1) PE(O−29:1) PE(O−30:1) PE(O−30:2) PE(O−31:1) PE(O−31:2) PE(O−32:0) PE(O−32:1) PE(O−32:2) PE(O−32:3) PE(O−33:1) PE(O−33:2) PE(O−33:3) PE(O−34:1) PE(O−34:2) PE(O−34:3) PE(O−34:4) PE(O−34:5) PE(O−34:6) PE(O−35:2) PE(O−35:3) PE(O−35:4) PE(O−35:5) PE(O−35:6) PE(O−36:1) PE(O−36:2) PE(O−36:3) PE(O−36:4) PE(O−36:5) PE(O−36:6) PE(O−36:7) PE(O−37:2) PE(O−37:3) PE(O−37:4) PE(O−37:5) PE(O−37:6) PE(O−37:7) PE(O−38:1) PE(O−38:2) PE(O−38:3) PE(O−38:4) PE(O−38:5) PE(O−38:6) PE(O−38:7) PE(O−38:8) PE(O−39:2) PE(O−39:4) PE(O−39:5) PE(O−39:6) PE(O−39:7) PE(O−39:8) PE(O−40:2) PE(O−40:3) PE(O−40:4) PE(O−40:5) PE(O−40:6) PE(O−40:7) PE(O−40:8) PE(O−40:9) PE(O−41:5) PE(O−41:6) PE(O−41:7) PE(O−41:8) PE(O−42:2) PE(O−42:3) PE(O−42:4) PE(O−42:5) PE(O−42:6) PE(O−42:7) PE(O−42:8) PE(O−42:9) PE(O−42:10) PE(O−43:7) PE(O−44:3) PE(O−44:5) PE(O−44:6) PE(O−44:7) PE(O−44:8) PE(O−46:7) PE(O−46:8)
label
Controle
Patient1
